# *NAXE* gene mutation-related progressive encephalopathy: A case report and literature review

**DOI:** 10.1097/MD.0000000000027548

**Published:** 2021-10-22

**Authors:** Li-Wei Chiu, Sheng-Shing Lin, Chieh-Ho Chen, Chien-Heng Lin, Ni-Chung Lee, Syuan-Yu Hong, I-Ching Chou, Chien-Lin Lin, Pei-Yu Yang

**Affiliations:** a China Medical University Hospital, Taichung, Taiwan; b Division of Pediatric Neurology, China Medical University Children's Hospital, Taichung, Taiwan; c Division of Pediatric Pulmonology, China Medical University Children's Hospital, Taichung, Taiwan; d School of Medicine, China Medical University, Taichung, Taiwan; e Department of Pediatric, National Taiwan University Hospital, College of Medicine, National Taiwan University, Taipei, Taiwan; f Department of Medical Genetics, National Taiwan University Hospital, College of Medicine, National Taiwan University, Taipei, Taiwan; g Graduate Institute of Integrated Medicine, College of Chinese Medicine, China Medical University, Taichung, Taiwan; h Department of Physical Medicine and Rehabilitation, China Medical University Hospital, Taichung, Taiwan.

**Keywords:** case report, early onset, NAD(P)HX epimerase gene, neurometabolic disorder, progressive encephalopathy

## Abstract

**Rationale:**

Progressive encephalopathy with brain edema and/or leukoencephalopathy-1 is an infantile, lethal neurometabolic disorder caused by a NAD(P)HX epimerase (NAXE) gene mutation. It is characterized by a fluctuating disease course with repeated episodes of improvement and regression. In this report, we present a rare case of NAXE gene mutation-related encephalopathy with unexpected neurological recovery and long survival time.

**Patient concerns:**

A 20-month-old girl presented with progressively unsteady gait and bilateral hand tremors after a trivial febrile illness. Her disease rapidly progressed to consciousness disturbance, 4-limb weakness (muscle power: 1/5 on the Medical Research Council scale), and respiratory failure. The patient gradually recovered 2 months later. However, another episode of severe fever-induced encephalopathy developed 2 years after the initial presentation.

**Diagnoses:**

Results of laboratory investigations, including complete blood count, blood chemistry, inflammatory markers, and cerebral spinal fluid analysis were unremarkable. Electroencephalography and nerve conduction velocity studies yielded normal results. Brain magnetic resonance imaging on diffusion-weighted imaging revealed abnormal sysmmetric hyperintensity in the bilateral middle cerebellar peduncles. A genetic study using whole exome sequencing confirmed the diagnosis of *NAXE* gene mutation-related encephalopathy.

**Interventions:**

Pulse therapy with methylprednisolone, intravenous immunoglobulin, coenzyme Q10, and carnitine were initially introduced. After a *NAXE* gene defect was detected, the vitamin B complex and coenzyme Q10 were administered. A continuous rehabilitation program was also implemented.

**Outcomes:**

*NAXE* gene mutation-related encephalopathy is usually regarded as a lethal neurometabolic disorder. However, the outcome in this case is better than that in the previous cases. She showed progressive neurological recovery and a longer survival time. The muscle power of the 4 limbs recovered to grade 4. At present (age of 5.5 years old), she can walk with an unsteady gait and go to school.

**Lessons:**

Although *NAXE* gene mutation-related encephalopathy is rare, it should be considered as a differential diagnosis of early onset progressive encephalopathy.

## 1 Introduction

Progressive encephalopathy with brain edema and/or leukoencephalopathy-1 (PEBEL-1) is a rare metabolic and autosomal recessive disorder that results from a NAD(P)HX epimerase (*NAXE*) gene mutation. Only 18 cases of this disorder have been reported.

**Figure 1:**
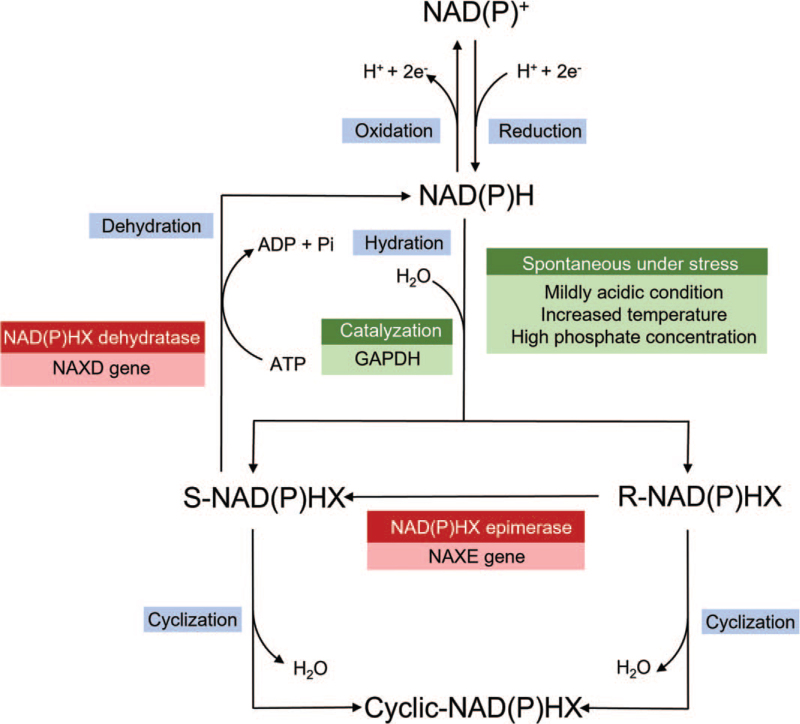
NAD(P)HX Repair System. ADP = adenosine diphosphate, ATP = adenosine triphosphate, e- = electron, GAPDH = glyceraldehyde-3-phosphate dehydrogenase, H+ = hydrogen cation, H_2_O = dihydrogen oxide, NAXD = NAD(P)HX Dehydratase, Pi = phosphate group.

Nicotinamide adenine dinucleotide and nicotinamide adenine dinucleotide phosphate are central to catabolic and anabolic reactions, biosynthetic pathways, and cellular antioxidant protection.^[1–5]^ The reduced forms of nicotinamide adenine dinucleotide and nicotinamide adenine dinucleotide phosphate are NADH and NADPH, respectively, which act as major redox equivalents in the mitochondrial electron transport chain. NADHX and NADPHX generated from the hydration of NADH and NADPH, respectively, are considered toxic metabolites that inhibit several important enzymes of the mitochondria.^[2,3,6]^*NAXE* gene, formerly known as APOA1BP (OMIM: 608862), encodes *NAXE*, which prevents the accumulation of toxic metabolites (Fig. 1).^[1,2,5]^*NAXE* gene mutations lead to the insufficiency of this repair system, as well as mitochondrial dysfunction, and therefore contributes to the accumulation of toxic metabolites that inhibit crucial enzymes.^[1,3]^ Most patients have a fluctuating disease course and then rapidly deteriorate and die within 2 years.^[1–4]^

This case report presents a case of *NAXE* gene mutation who presented with encephalopathy with a fluctuating disease course. *NAXE* gene mutation-related encephalopathy is usually regarded as a lethal neurometabolic disorder. However, the outcome in this case was better than that in the previous cases. She showed progressive functional recovery and a longer survival time. The clinical course, characteristics, laboratory and imaging findings, and treatments for *NAXE* gene mutation-related encephalopathy will be discussed in this report.

## 2 Case presentation

A 20-month-old girl without systemic history presented with progressively unsteady gait and bilateral hand tremors for 5 days after a trivial febrile illness. The fever persisted for 5 days before admission and subsided temporarily after the administration of antipyretics. General weakness, decreased appetite, productive cough, and rhinorrhea were noted. Previously acquired motor milestones, including sitting, rolling, standing, and walking, were lost in 5 days. She had a sick contact with her mother, who had upper respiratory infection syndrome 7 days before hospitalization. The patient had no history of allergy, prior episodes of neurological deficits, travel, trauma or exposure, no current medication, or recent immunization. The girl was born to healthy non-consanguineous parents after an uneventful pregnancy at 38 weeks of gestational age with a birth weight of 2850 g. She had normal development milestones with uneventful neonatal and infantile periods until this episode. The patient had no family history of metabolic, neurological, vascular, hematological, or autoimmune diseases. Upon physical examination in the emergency department, she had mild fever (body temperature, 37.3°C), was unable to sit, stand, and walk, and had incoordination when holding objects. The patient was admitted for further evaluation.

**Figure 2:**
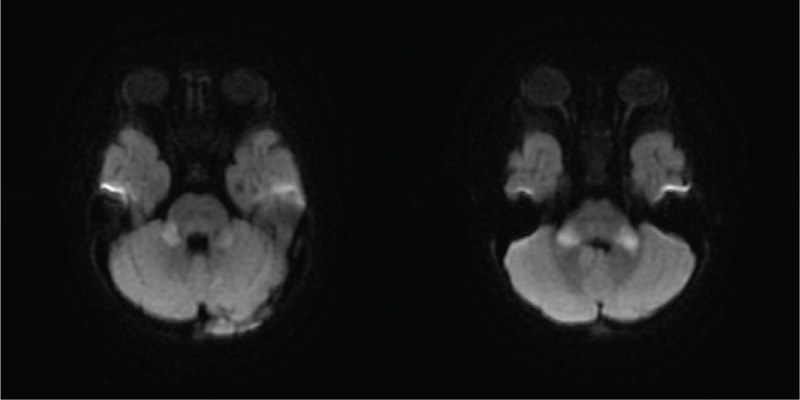
Brain magnetic resonance imaging (MRI) revealed abnormal symmetric hyperintensity in the bilateral middle cerebellar peduncles on diffusion-weighted imaging (DWI).

The results of complete blood count with differential count and platelet count, serum electrolytes, blood glucose, coagulation studies, serum aminotransferases, blood urea nitrogen, creatinine, inflammatory markers, and microbiological tests (blood culture, herpes simplex virus polymerase chain reaction, and enterovirus polymerase chain reaction) were unremarkable. A nerve conduction velocity test, electroencephalography (EEG), and cerebrospinal fluid (CSF) analysis did not reveal any abnormalities. Brain magnetic resonance imaging (MRI) (Fig. 2) revealed abnormal symmetric hyperintensity in the bilateral middle cerebellar peduncles on diffusion-weighted imaging (DWI). Pulse therapy with methylprednisolone (30 mg/kg/day for 5 days) and intravenous immunoglobulin (IVIG) were introduced, but her condition remained unchanged.

**Figure 3:**
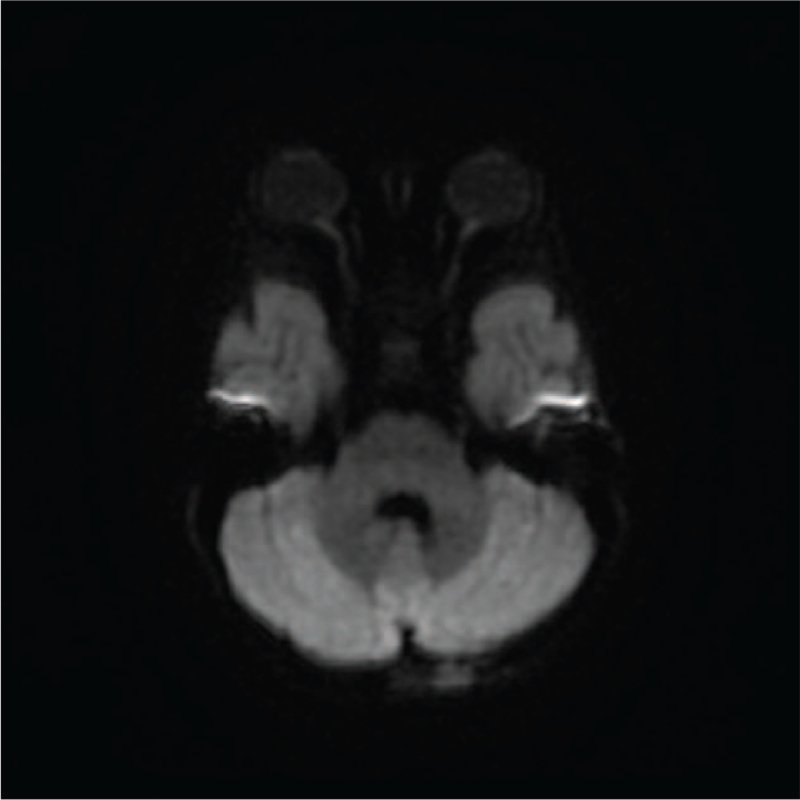
Brain magnetic resonance imaging (MRI) demonstrated complete remission of lesions in the bilateral middle cerebellar peduncles on diffusion-weighted imaging (DWI).

Ten days after the initial presentation, the disease rapidly deteriorated with fever, progressive consciousness disturbance, 4-limb weakness (muscle power: 1/5 in 4 limbs on the Medical Research Council scale [MRC] scale), and dysphagia. The Glasgow Coma Scale (GCS) score was E2M4V2. The disease rapidly progressed to coma and respiratory failure. Emergency endotracheal intubation was performed. According to the onset of encephalopathy preceding a febrile illness with a fulminant clinical course and rapid deterioration, acute necrotizing encephalopathy of childhood, acute disseminated encephalomyelitis, mitochondrial disease, autoimmune encephalitis, and other metabolic disorders were suspected. Brain MRI demonstrated complete remission of lesions in the bilateral middle cerebellar peduncles on DWI comparing with the previous MRI (Fig. 3). However, there were residual abnormal hyperintense lesions in the cerebellar tonsil, bilateral cerebellar hemispheres, superior cerebellar peduncles, and posterior aspect of the midbrain on DWI and T2 weighted image. EEG revealed general slowing activity. The results of CSF analysis, autoimmune work-up, metabolic panels, heavy metal test, toxicity screening, thyroid function test, serum cortisol level, tumor markers, and abdominal ultrasonography were unremarkable. The correct diagnosis was not yet made at that time, but we narrowed the probable diagnoses to mitochondrial disease, autoimmune disease, and other metabolic disorders. Therefore, ubiquinol (coenzyme Q10) for ten days, carnitine for ten days, pulse therapy with methylprednisolone, and IVIG were prescribed empirically.

The patient remained comatose with ventilation during the first month. Bedside rehabilitation programs, including passive range of motion and proper positioning, have been introduced since then. The endotracheal tube was successfully removed and shifted to bilevel positive airway pressure in the second month. The inpatient rehabilitation program was adjusted according to the patient's condition. As her neurological condition improved gradually, a more aggressive program including active range of motion exercise, bed mobility exercise, sitting balance training, and swallowing training was introduced. After 2 months of hospitalization, the patient was discharged with gradual clinical improvement. Her consciousness level was clear (GCS: E4M6V5). She was able to sit and crawl, but still could not stand and walk. Residual deficits included bilateral hemiparesis (muscle power: 3/5 in 4 limbs on the MRC scale), dysphagia, and global development delay.

The patient underwent a rehabilitation program for 2 years. Improvement in motor function (muscle power: 4/5 in 4 limbs on the MRC scale), swallowing ability, trunk control, and speech were noted. She was able to walk independently for a short distance with an unsteady gait. Her condition gradually improved.

**Figure 4:**
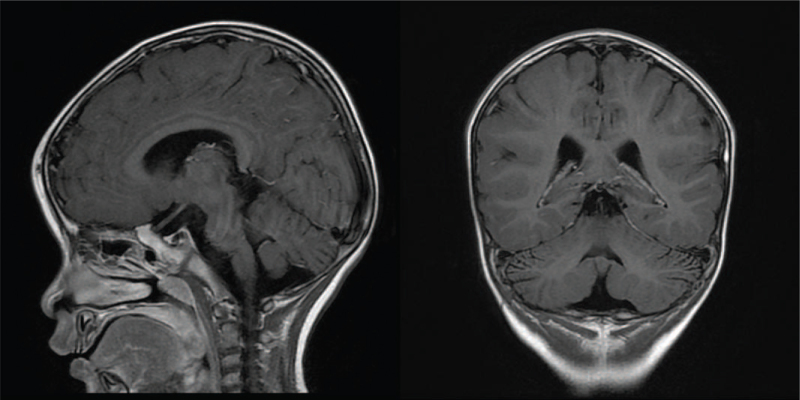
Brain magnetic resonance imaging (MRI), showing subtle sulcal widening of bilateral cerebellar hemispheres and enlargement of the fourth ventricle reflecting brain tissue loss in T1 weighted image (T1WI).

She developed another episode of acute deterioration 2 years after her initial presentation. She suffered from progressive ataxia with anisocoria after a fever episode. Chest radiography revealed mild bronchopneumonia. Brain MRI revealed no apparent abnormal enhancement except for subtle sulcal widening of the bilateral cerebellar hemispheres and enlargement of the fourth ventricle reflecting brain tissue loss (Fig. 4). She presented with bilateral esotropia, and abducens nerve palsy was suspected. Ataxia and esotropia improved after IVIG administration. Five days later, the disease rapidly progressed with fever, bilateral upper extremity tremor, bilateral esotropia, consciousness disturbance (GCS: E2M2V2), and 4-limb weakness (muscle power: 0/5 in 4 limbs on the MRC scale). She was intubated and received cardiopulmonary resuscitation because of oxygen desaturation, severe respiratory acidosis, and bradycardia. A series of evaluations, including cardiac ultrasonography, brain computed tomography, and spine MRI, were performed, but no abnormalities were found. Mannitol was used for increased intracranial pressure. Clozapine and gabapentin were prescribed for simple partial seizures. Metabolic or autoimmune disorders were still highly suspected. A genetic study was conducted because of the unusual clinical course. Whole exome sequencing revealed defects in the APOA1BP gene, also known as *NAXE* gene, which produces apolipoprotein A-I-binding protein catalyzing the epimerization of the S- and R-forms of NAD(P)HX in apolipoprotein A-I.^[1]^ Impairment of the APOA1BP gene results in cholesterol metabolism disorders and causes progressive early onset encephalopathy with brain edema and/or leukoencephalopathy.^[1]^

The patient had a defect in the APOA1BP gene with chr.1:156563742 A>C/C, RefSeq NM_144772 c.A733C, p.K245Q. She had a homozygous c. 733A>C mutation in the APOA1BP gene, which led to the substitution of lysine with glutamine. The K245Q variant affected the structure of the secondary protein, resulting in NAD(P)HX epimerase dysfunction.^[7]^ Vitamin B complex (thiamine 60 mg, riboflavin 3 mg, nicotinamide 30 mg and pyridoxine 3 mg per day) for 14 days and coenzyme Q10 were administered. She was extubated and changed to bi-level positive airway pressure in the third month of hospitalization. The inpatient rehabilitation program was initiated soon after she was admitted to the hospital and was adjusted according to her clinical condition. The program involved a multidisciplinary team approach including medical management, physical therapy, occupational therapy, speech therapy, respiratory management, nutritional services, social workers, and assistive device prescription. She had clear consciousness (GCS: E4M6V5) but still had bilateral hemiplegia (muscle power: 2/5 in 4 limbs on the MRC scale), dysphagia with nasogastric tube feeding, spasticity over 4 limbs, poor sitting balance, and aphonia at the time of discharge. She can breathe spontaneously without ventilator support.

In the following years, she was in a stable condition and gradually improved. She was not taking any medications. She underwent a continuous rehabilitation program including physical therapy, occupational therapy, and speech therapy. Her speech, swallowing, motor function, and cognitive function improved gradually. We followed up for 3 years and 9 months after onset. At present (age of 5.5 years old), she can walk with an unsteady gait for short distances, communicate with short sentences and dysarthric voice, and use spoon for feeding. The muscle power of the 4 limbs recovered to grade 4. Her full intellectual quotient was 75, as tested by the Wechsler Preschool and Primary Scale for Intelligence. At the age of 5 years, she started schooling, and her family reported that she could participate in school activities under moderate assistance.

## 3 Discussion

Neurological deterioration of this rare genetic disorder is triggered by a febrile illness. A previous study even reported a case triggered by tetrahydrocannabinol and alcohol.^[3]^ This infantile, lethal neurometabolic disorder has a fluctuating disease course with repeated episodes of improvement and regression. This leads to rapid deterioration and death in early childhood. The predominant clinical features include cerebellar ataxia, muscular hypotonia, gait difficulty, delayed developmental milestones, seizures, respiratory failure, and conscious disturbance (Table 1).^[1–4]^

**Table 1:** Clinical characteristics of progressive encephalopathy with brain edema and/or leukoencephalopathy-1(PEBEL-1).^[1–7]^.

	Number of occurrences (Total cases: 19)
Male: female	13:6
Onset age	≤1 y/o: 71–2 y/o: 72–3 y/o: 1>3 y/o: 2Unknown: 2
Survival time	≤1 y/o: 71–2 y/o: 52–3 y/o: 1Still alive when the study was published: 3Unknown: 3
Muscular hypotonia	10
Delayed developmental milestone	10
Respiratory failure	10
Conscious disturbance	9
Seizure	6
Nystagmus	5
Gait difficulty	5

Laboratory investigations, including complete blood count, biochemistry, serum electrolytes, blood glucose, coagulation studies, serum aminotransferases, blood urea nitrogen, creatinine, inflammatory markers, and microbiological tests, usually revealed normal results that are consistent with the present case. Kremer et al reported 2 cases with elevated serum lactate and 1 case with abnormal liver aminotransferase levels.^[2]^ The results of CSF analysis were documented in 14 cases. Seven cases, including our case, showed normal results.^[1,3]^ However, seven cases showed a high level of lactate.^[2,3]^

The brain MRI findings varied among the 19 cases. Hyperintense white matter signal in T2 and/or DWI was seen in 10 cases, including our case.^[1,2,4]^ Nine cases had normal brain MRI initially.^[1–3]^ Brain atrophy was reported in 4 cases.^[2,3]^ Four cases showed brain edema, which is consistent with our case.^[2]^ Other brain MRI abnormalities of this case that had been reported in previous cases include hyperintensity in the middle cerebellar peduncles reported by Kremer et al^[2]^ and ventricle enlargement reported by Incecik et al^[4]^ Trinh et al reported a case with normal spine MRI results, which is in line with our case.^[3]^ Myelopathy in spine MRI was shown in 3 cases reported by Kremer et al^[2]^

EEG results were reported in nine patients. Eight cases, including our case, demonstrated slow activity.^[1,2]^ Epileptic discharge was noted in 1 case.^[4]^

The results of laboratory investigations, image studies, and EEG were non-specific. The diagnosis of *NAXE* gene mutation-related encephalopathy is based on the characteristic clinical course and results of a genetic study. Previous cases mostly had disease onset at 1–2 years of age and died within 2 years after the initial presentation (Table 1). Patients mostly received intubation and mechanical ventilation support, and eventually died. The few patients who endured were left with everlasting deficits and resulted in a bedridden or vegetative state with ventilator support. Among the 19 reported cases, including our case, only 3 cases were alive when the study was published.^[1–4,8]^ Among the 3 survivors, one had a disease onset at 6 to 12 months and was alive at 5.5 years old but required a mechanical ventilator.^[1]^ Another case had a disease onset at 22 years and she was still alive at 29 years of age without tracheotomy but remained bound to a wheelchair.^[3]^ Several antiepileptic drugs, vitamin B3, and coenzyme Q10 were administered to stabilize her clinical course. Residual deficits included perioral dyskinesias, divergent strabismus, mild limb spasticity, and neuropathic pain. However, the outcome in our case was better than that in the previous cases. She had a disease onset at 20 months of age and survived the disease at 5.5 years old with stable condition and unexpected partial recovery. After the latest admission, she did not undergo any treatment other than rehabilitation. She showed considerable neurological improvement, including speech, motor, and cognitive functions. Compared with other cases, this case had a similar onset age and typical disease course but had a longer survival interval and better outcome.

Due to the small number of case reports, the discussion about the optimal treatment of PEBEL-1 is limited. Vitamin B3 and coenzyme Q10 are treatment options for consideration.^[3]^ Trinh reported positive results after administration of a combination of vitamin B3 and coenzyme Q10. Avoidance of trigger factors, combination of vitamin B3 and coenzyme Q10, and administration of antiepileptic drugs have been reported to contribute to a positive outcome. In our case, antiepileptic drugs, vitamin B complex, and coenzyme Q10 were administered for a short time during hospitalization. She did not receive any medications during the recovery phase. Therefore, the possibility of spontaneous improvement must be considered. However, the optimal treatments and prognostic factors need to be studied further.

## 4 Conclusion

This case report aimed to discuss the clinical course and characteristics of PEBEL-1. The data presented may help specialists in making early diagnoses to enable early treatment. The clinical course, laboratory data, imaging findings, and effective treatments remain inconclusive because few cases have been reported. Further studies are needed to explore the diagnosis and treatment of this rare disease. Although *NAXE* gene mutation-related encephalopathy is rare, it should be considered as a differential diagnosis of early onset progressive encephalopathy.

## Author contributions

**Conceptualization:** Li-Wei Chiu, I-Ching Chou, Pei-Yu Yang.

**Data curation:** Li-Wei Chiu, Chieh-Ho Chen, Chien-Heng Lin, Ni-Chung Lee, Syuan-Yu Hong, Pei-Yu Yang.

**Formal analysis:** Sheng-Shing Lin, Chieh-Ho Chen, Chien-Heng Lin, Syuan-Yu Hong, Pei-Yu Yang.

**Investigation:** Sheng-Shing Lin, Chieh-Ho Chen, Chien-Heng Lin, Ni-Chung Lee, I-Ching Chou, Pei-Yu Yang.

**Methodology:** Syuan-Yu Hong, Pei-Yu Yang.

**Project administration:** Li-Wei Chiu, Pei-Yu Yang.

**Resources:** I-Ching Chou, Chien-Lin Lin, Pei-Yu Yang.

**Supervision:** Sheng-Shing Lin, I-Ching Chou, Chien-Lin Lin, Pei-Yu Yang.

**Validation:** Chien-Heng Lin, Ni-Chung Lee.

**Visualization:** Chieh-Ho Chen, Pei-Yu Yang.

**Writing – original draft:** Li-Wei Chiu, Pei-Yu Yang.

**Writing – review & editing:** Li-Wei Chiu, Sheng-Shing Lin, Pei-Yu Yang.
